# The Epizooty at San Francisco

**Published:** 1881-10

**Authors:** W. H. Carpenter

**Affiliations:** 966 Howard St., San Francisco, Cal.


### II.—THE EPIZOOTY AT SAN FRANCISCO—ITS TREATMENT, AND A THEORY OF ITS PATHOLOGY.

---

EDITOR OF JOURNAL OF COMPARATIVE MEDICINE:

One of the greatest difficulties with which the practitioner of veterinary medicine has to contend, is the treatment of that class of diseases termed epizootic. In no other class of cases is the superiority of the well-educated veterinary surgeon over the empiric better exemplified than in the manner in which this description of malady is treated by each. Here the skill of the empiric generally falls far short of the great expectations that have been raised by his extensive experience and long catalogue of former cures. For, unfortunately for his reputation, and the pockets of those proprietors of animals requiring his aid, the disease, although so extensive in its character, so rapid in its course, and so fatal in its termination, is yet generally so transitory in its sojourn, that he cannot profit by that experience which is the only thing to guide him in the practice of his art. It is generally when the disease has almost entirely disappeared, when such knowledge is no longer of any practical utility, that he commences to understand anything about its peculiarities. Dearly bought is such experience; for perhaps, by the exhibition of improper remedies, he has been the cause of death to the majority of his patients, when in many cases, were nature left to herself, her powers would have triumphed, and the animals recovered.

There has been a disease prevalent for some months past among the horses of this country, and there has been a number of fatal cases, due largely, I think, to an injudicious mode of treatment, resulting from ignorance of the true pathology of the affection. A thorough knowledge of epizootic diseases is one of the most important parts of veterinary education, for they are diseases which will occasionally destroy, in a short time, great numbers of useful animals; their causes are obscure or concealed, their march insidious and rapid.

Epizootic diseases are ordinarily transmitted with extreme facility from one individual to another; they generally present themselves under the same aspect, following one analogous march, offering occasionally anomalies, the just value of which are not appreciated, or which are uselessly magnified into particular species.

It seems to be a law in the animal economy that the action of an organ or set of organs cannot be increased without that of others being proportionately diminished. This law appears to extend even to the nervous system. For example, there may be an increased action going on in the organic nerves or system of the sympathetic, with which the mucous membranes are so plentifully supplied. There is then present, as a consequence, a proportionate degree of debility in the motor system, producing that intense prostration of strength always observed in extensive epidemic affections of the mucous membranes. The contrary effect may be observed to take place when there is a morbid increase of action in the motor system, or those nerves supplying the muscles of volition. Tetanus offers a most striking and familiar example of this. Here there is an increase of irritability of the spinal cord, so that all the muscles of volition are thrown into intense activity. The organic system then becomes greatly debilitated, loses in a measure its influence, and as a result there is such a torpid state of the digestive organs, that in many cases the most drastic medicines fail to produce the slightest effect on the intestinal mucous membrane.

These facts throw considerable light on the kind of treatment required to be employed in the present "epizootic" among horses. It is evidently a constitutional affection, exerting its influence principally on the organic system of nerves, through the medium of which it affects the mucous and tegumental membranes generally, more particularly those that have been already rendered peculiarly predisposed by chronic disease or some peculiar idiosyncracy of the individual. In confirmation of this assertion, it may be adduced that when there is a disposition to ophthalmia, the affection of the eyes is sure to be more intense, particularly in the one before affected; and where there is a tendency to diarrhœa, the mucous membrane of the intestines suffers more than any other part of the animal; also, that in cases of chronic bronchial irritation the respiratory system suffers much more considerably than under any other circumstances. We must entirely discard the idea of its being merely a simple inflammatory affection of one or more of the mucous membranes, with fever occasionally as an accidental attendant. The results of treatment suggested by such an idea have proved its erroneousness beyond a doubt. By bleeding, you only diminish the already far too weakened forces of the animal, without at all striking at the root of the evil. Give the ordinary dose of aloes, and you produce super-purgation. The bronchial membrane also sympathizes, and you thus establish what is a thousand-fold more difficult to contend with than the original disease, which will, like all epizootical diseases when once set in, run its prescribed course.

In treatment some agent is required, the action of which will have the effect of allaying the increased irritability of the organic system, and thus, by striking at the fountain-head of diseased action, remove those derangements of function which, although properly speaking are merely secondary effects, are by many considered as the primary element of the disease. For this purpose I have had recourse, and with the happiest effects, to the potassio-tartrate of antimony, a medicine which possesses the valuable qualities of acting on the mucous tissues, not alone by mere contact, but more particularly through the medium of the nerves by becoming absorbed into the system and thus acting on their centres. It also has the effect of allaying increased arterial action. When given repeatedly it acts as an aperient, and where the bronchial mucous membrane is threatened or has already become implicated, there is no remedy better calculated to avert the danger. In some cases I have combined this medicine, and I have thought advantageously, with a little of the nitrate of potass—the dose of the tartrate, three and a half drachms, and that of the nitrate, two drachms—given morning and evening dissolved in a little water. By giving them in water their action is almost immediate, and the effect is better than when given in balls. The tartarized antimony, when given in its solid state, is very liable to irritate the stomach, and in man causes vomiting. The horse is, however, a most admirable subject for the exhibition of such an agent, he being an animal to whom the power of vomiting is denied. Nausea can, therefore, in his case be carried almost to any extent. When an animal is first observed to be affected with the "epizootic," it is desirable to place him in a large box-stall, allowing him to have as much air as possible, both throughout the day and during the night. Of air, no matter how cool the temperature, he cannot possibly have too much. This is an axiom that should never be forgotten by the veterinary practitioner. The animal should, however, be well clothed and the legs bandaged, but not too tightly, with flannel. Such means have a tendency to equalize the circulation, particularly where there is diminution of heat. Let one of the principal objects be to allow the patient to respire plenty of pure air, at the same time keeping the surface of the body moderately warm, if the season be at all inclement.

I am well aware that there are a number of veterinary practitioners, and some having pretensions to eminence, whose treatment consists in copious bleeding, blistering the throat and sides, and then turning their patients into a field or paddock, regardless of the weather, there to live or die as chance may decree. To any person of common sense, at all acquainted with medical matters, such a mode of treatment must appear in its true light—a lamentable example of the bigoted ignorance which still remains among many members of the veterinary profession.

W. H. Carpenter, V.S.

966 Howard St., San Francisco, Cal.

